# The Treatment In Morning versus Evening (TIME) study: analysis of recruitment, follow-up and retention rates post-recruitment

**DOI:** 10.1186/s13063-017-2318-4

**Published:** 2017-11-23

**Authors:** David A. Rorie, Robert W. V. Flynn, Isla S. Mackenzie, Thomas M. MacDonald, Amy Rogers

**Affiliations:** 1 Division of Molecular and Clinical Medicine, University of Dundee, Ninewells Hospital and Medical School, Dundee, DD1 9SY UK; 2 Medicines Monitoring Unit, University of Dundee, Ninewells Hospital and Medical School, Dundee, DD1 9SY UK

## Abstract

**Background:**

The use of information technology (IT) is now the preferred method of capturing and storing clinical research data. The Treatment In Morning versus Evening (TIME) study predominantly uses electronic data capture and IT to compare morning dosing of hypertensive medication against evening dosing. Registration, consent, participant demographics and follow-up data are all captured via the study website. The aim of this article is to assess the success of the TIME methodology compared with similar studies.

**Methods:**

To assess the TIME study, published literature on similar clinical trials was reviewed and compared against TIME recruitment, follow-up and email interaction data.

**Results:**

The TIME website registered 31,695 individuals, 21,116 of whom were randomised. Recruitment cost per randomised participant varied by strategy: £17.40 by GP practice, £3.08 by UK Biobank and £58.82 for GoShare. Twelve-month follow-up retention rates were 96%. A total of 1089 participants have withdrawn from their assigned time of dosing, 2% of whom have declined follow-up by record linkage or further contact. When the TIME data are compared with similar study data, study recruitment is very successful. However, TIME suffers difficulties with participant follow-up and withdrawal rates similar to those of conventional studies.

**Conclusions:**

The TIME study has been successful in recruitment. Follow-up, retention rates and withdrawal rates are all acceptable, but ongoing work is required to ensure participants remain engaged with the study. Various recruitment strategies are necessary, and all viable options should be encouraged to maintain participant engagement throughout the life of studies using IT.

## Background

Randomised controlled trials are the most reliable method of evaluating the efficacy or safety of a drug therapy [1]. Research and clinical studies have evolved to the use of the newest technologies and methodologies, with electronic data capture (EDC) now being commonplace and preferable [2–5]. In parallel, email use has quickly developed to replace face-to-face consultations [6–8] and study screening visits [9]. Email has also been shown to be an effective intervention technique for changing participant behaviour [10–13] and an effective method of recruitment to clinical trials [14]. Further research is needed to understand how studies that use information technology (IT) compare with similar conventional studies in terms of efficiency and validity.

The Treatment In Morning versus Evening (TIME) study [15] is a British Heart Foundation (BHF)-funded clinical study comparing morning dosing of usual hypertensive medication with evening dosing to determine if one is better than the other in terms of cardiovascular outcomes. The study is conducted predominantly using an online website and is an example of a study where IT and EDC are used to maximise study proficiency and minimise costs.

The TIME study uses a novel online methodology and IT to recruit participants, obtain consent, collect baseline data and follow participants. Interested individuals self-enrol on the study website (www.timestudy.co.uk) with a password of their choice and their email address. Those who choose to take part then consent and enter demographic data via the secure website, are followed at routine intervals via automated emails, and are asked to enter patient-reported outcomes (PROs) online. The study has surpassed recruitment expectations, with over 21,000 participants randomised over a 2-year period. However, it is unclear how successful the study methodology will be compared with conventional data collection methods. The purpose of this research paper is to evaluate the TIME study and its online methodology in each of the following areas:

- Recruitment
- Follow-up, retention and adherence
- Email and telephone interactions

## Methods

**Fig. 1:**
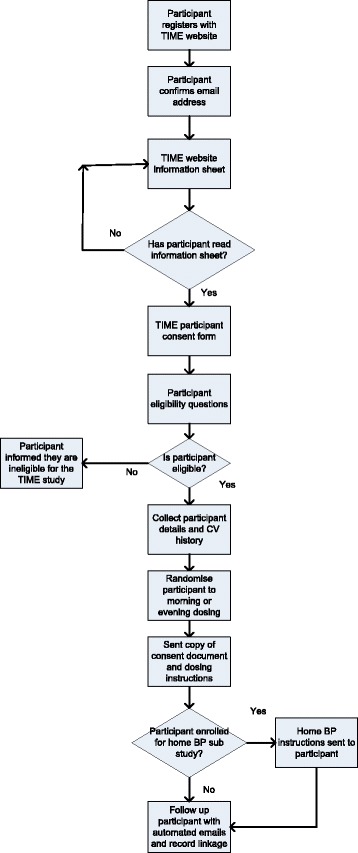
Flow diagram for Treatment In Morning versus Evening (TIME) study website. *BP* Blood pressure, *CV* Cardiovascular

The TIME study recruited participants using various recruitment strategies: radio advertising, posters in general practitioners’ (GPs’) practices, newspapers, direct email and letters from GPs, hospital clinics, pharmacies, and research study databases of individuals who had agreed to be contacted about research. Interested individuals were invited to register on the study website, to submit how they heard about the study, indicate their consent to participate, and enter the demographic information and medical history data necessary for the study (Fig. 1). Participants were also asked to provide contact information for a nominated surrogate who could be contacted in the event that email contact with the participant was lost. A copy of the participant’s consent form was sent to the participant along with an email confirming their allocated time to take their hypertensive medication (morning or evening). Follow-up emails are sent on a 3-monthly basis to assess adherence to allocated dosing time and to identify any potentially related adverse events that the participant has experienced. Owing to the potentially poor quality of data from PROs [16], record linkage to nationally held hospitalisation and mortality data is used to identify potential study endpoint events. Participants are able to contact study staff at any point during the study using a Freephone number and by email or post. Data on study contact by participants were collected during active recruitment by GP practices and after recruitment by GP practices had ceased, in order to assess resource use during the recruitment phase of the study.

To evaluate the study methodology, a review of published literature was conducted. The PubMed library was searched using general search terms with the aim of identifying average cost per randomised study participant, retention rates, rates of loss to follow-up, withdrawal rates, and email use and interactions in clinical trials. The remainder of this article compares the TIME study data with the study data identified from the literature review, highlighting the studies’ successes and limitations as well as what should be investigated in further research.

## Results

The TIME study began as a feasibility pilot [17] in December 2011. On the basis of data derived from the pilot study, the BHF funded TIME to recruit 10,269 participants into a formal study. To accommodate the decreasing cardiovascular event rate observed in other studies [18], the recruitment target was increased to at least 21,000. It was believed that doubling the recruitment target would ensure achieving the necessary number of events needed to power the study within a reasonable time frame. This increase was made possible by the success of the various recruitment strategies used.

### Recruitment

**Fig. 2:**
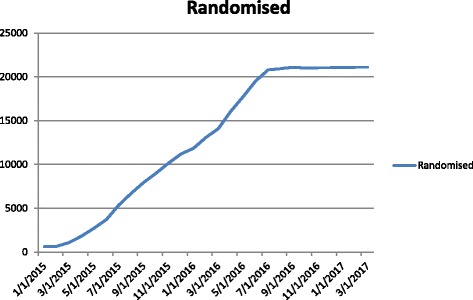
Treatment In Morning versus Evening (TIME) study recruitment of randomised participants from 2015 to 2017 (*n* = 21,116)

Active recruitment into the study closed in August 2016, and the TIME study website closed to new registrations at the end of March 2017. The majority of participants were randomised between March 2015 and July 2016 (20,132 [95%]), largely due to the work of clinical research networks and GP practices in sending letters to patients with hypertension. During this period, the most successful month was April 2016, in which 1989 participants were randomised. The least successful month, March 2015, saw 421 participants randomised. On average, TIME randomised 1184 participants per month over this period. Overall, between the pilot start date, December 2011, and the registration closure date, August 2017, 31,695 individuals registered with the TIME study website. Of these, 23,617 (72%) met the eligibility criteria (≥18 years old, not currently in a study, having a valid email address, and receiving one or more antihypertensive drugs) and went on to complete the online consent process. A total of 2502 (8%) individuals consented, and were eligible, but did not complete the enrolment process and go on to be randomised into the study. A total of 21,116 (67%) individuals went on to be randomised into the study and were allocated a time of dosing for their antihypertensive medication (Fig. 2).

The TIME study was advertised to potential participants using various strategies. One month after the pilot study began recruitment, data were captured on how registered visitors to the website had come to hear about the study. Each recruitment strategy varied in cost and effectiveness; this is detailed in Table 1. In an effort to expedite recruitment, UK Biobank [19] and GoShare [20] were recruited to assist in contacting individuals and making them aware of the study. UK Biobank and GoShare are both health registries whose aim is to improve the prevention, diagnosis and treatment of a wide range of serious and life-threatening illnesses. These databases contain details on individuals who have voluntarily registered, given detailed information about themselves and agreed to have their health status tracked. Over many years these will build into powerful resources to help scientists discover why some people develop particular diseases and others do not. UK Biobank sent 79,628 emails to participants with hypertension in their database at a total cost of £10,000, or £3.08 per randomised participant (recruitment rate of 4.1%). GoShare sent 12,931 emails to patients with hypertension at a cost of £2000, or £58.82 per randomised participant. The largest number of TIME participants were recruited from GP practices that had sent individual letters inviting patients to register on the study website. GP practices sent 594,289 letters to patients known to be on at least one antihypertensive medication, using the Docmail [21] mailing service, at a cost of £277,140, or £17.40 per randomised participant (recruitment rate of 2.7%). Posters displayed in primary and secondary care settings advertising the study were not as successful [22], initially costing £315 per randomised participant. This cost has been reduced to £78 per randomised participant as a result of the work of the research networks engaging with practices and distributing additional posters during the recruitment period. Other recruitment strategies used were either free or conducted and funded by supporting bodies, including the BHF, the British and Irish Hypertension Society, the National Institute for Health Research (NIHR) Clinical Research Network, and the Scottish Primary Care Research Network.

**Table 1:** Treatment In Morning versus Evening (TIME) study registration statistics: how participants heard about the study and cost, if known

Recruitment method	Registrations (*n* = 30,775) (%)	Randomised (*n* = 20,914) (%)	Cost per randomised participant (if known)
Radio	70 (< 1)	62 (< 1)	
Friends/relatives	102 (< 1)	65 (< 1)	
Web search	96 (< 1)	53 (< 1)	
From GP	22,675 (74)	15,932 (76)	£17.40
MEMO website	104 (< 1)	44 (< 1)	
Practice poster	261 (< 1)	161 (< 1)	£78.00
TIME video (YouTube)	14(< 1)	6 (< 1)	
Medscape	8 (< 1)	3 (< 1)	
BHF Heart Matters	332 (1)	249 (1)	
Pharmacy	27 (< 1)	16 (< 1)	
Newspaper	17 (< 1)	8 (< 1)	
Hospital or clinic	939 (3)	615 (3)	
Biobank	5358 (17)	3242 (16)	£3.08
GoShare	55 (< 1)	34 (< 1)	£58.82
Other	717 (2)	424 (2)	

*BHF* British Heart Foundation, *GP* General practitioner

### Recruitment review

The literature identified suggests that researchers should use various methods to meet recruitment targets [23, 24]. Over the past decade participant recruitment in clinical trials has not increased, with only 3% of eligible participants agreeing to take part in clinical research [25]. The response rate to TIME study invitations was similar (4.1% for Biobank and 2.7% for GP letters), but the low-cost recruitment strategies implemented allowed for researchers to double the study population to 21,000 from the initial 10,269, despite research governance delays [26]. Involvement of the NIHR Clinical Research Network and the Scottish Primary Care Research Network was invaluable in achieving this.

Trial recruitment costs are reported to vary greatly between studies [27, 28]. Online recruitment has consistently been successful at recruiting more participants in terms of raw numbers and recruiting participants from hard-to-reach populations, and it is more cost-effective than offline methods [29]. However, the technology does bring new challenges. It has been suggested that online recruitment could hinder the identification of well-defined populations and trials reporting PROs have been met with some scepticism about reliability [30]. The TIME study researchers aimed to mitigate this by using PROs to augment a primary study endpoint determined by record linkage to hospitalisations and deaths. To ensure that online studies are generalisable to the target population, it will be important to assess whether participants recruited online are representative of the target population.

Direct contact with study staff is still preferable for some participants [31], and barriers other than household internet access still exist that need to be considered during the recruitment process [32]. During the TIME study recruitment, 8074 people registered but failed to consent or were ineligible, and 2502 individuals failed to complete the enrolment process. The reasons for this remain unknown, and owing to ethical restrictions we are unable to collect these data. Participants clearly responded positively to letters from their own GPs’ practices; this appears consistent with similar studies in the United Kingdom using equivalent recruitment strategies [33].

### Follow-up, retention and adherence

PROs are elicited in the TIME study by follow-up emails sent to the participants on a 3-monthly basis. Participants are asked to click on a link within each follow-up email, which automatically takes them to the study website, where they are asked to submit their data. As of March 2017, there were 96,418 completed follow-ups logged on the database. There were a mean of four follow-ups per randomised participant (median of five). The most follow-ups reported by a single participant were 25. Approximately 96% (19,882) of all live study participants (not withdrawn or deceased) had follow-up information available from within the last 12 months, and efforts are ongoing to contact those participants for whom follow-up data are overdue.

If participants fail to respond to their first follow-up email, they are sent a reminder follow-up email after 14 days. If, after a further 14 days, they fail to respond to the second follow-up email, then an email is sent to the participant’s surrogate whom they nominated during enrolment, asking after the well-being of the participant and for any reasons the participant may not have replied to study emails. A total of 6158 surrogate emails had been sent between March 2012 and March 2017, resulting in 669 (11%) responses from participant surrogates.

In an effort to capture whether participants had read and understood their allocated time of dosing email, a link was added to the allocation email. This link asked the participant to click it if they had understood and acknowledged the time they had been requested to take their hypertensive medication. This link was incorporated into the allocation email in October 2015, and 11,483 (54%) of randomised participants received the email link. Of these, 7949 (69%) participants clicked the link indicating they had received and understood their allocation email. A total of 3471 (16%) participants have indicated that they have not adhered to their allocated time of dosing during at least one 3-month follow-up period in the study. A total of 1089 people have formally withdrawn from email follow-up since the pilot began in 2011 (11,070,614 patient-days), but only 388 (2%) indicated refusal to be followed by record linkage.

### Follow-up, retention and adherence review

Whilst there are studies conducted on improving participant adherence [34–37], adherence to allocated treatment during clinical studies is under-reported in the literature. Drop-out rates and loss to follow-up are more widely documented. Reported drop-out rates for studies vary greatly, from 0% to 45.5% [38, 39]. Reasons for withdrawal are occasionally reported under typically generic categories, with ‘patient decision’ and ‘adverse event’ being the most frequently reported [40]. A suggested general ‘rule of thumb’ is that < 5% drop-out leads to little bias, whereas > 20% poses a serious threat to validity [41]. Currently 5% of participants have withdrawn from TIME. Participants who wish to withdraw from studies owing to being allocated to their unfavoured treatment arm is a concern and indicates a disappointing lack of understanding of the clinical research process, despite extensive pre-consent written information. Such disappointment is known to be common in clinical studies where participants had hoped to be allocated to an active treatment group. Consent information is of high importance because those who are very disappointed often claim they did not receive understandable information [42]. In addressing specific patient concerns, attention to a patient’s emotional status and expectations of trial participation have been found to be associated with better adherence [43]. Researchers in a study of cardiovascular events in diabetes (ASCEND) [33] implemented a run-in period before participants were randomised to ascertain protocol compliance. This process helped alleviate the drop-out rate by identifying non-compliant participants early in the study process and before randomisation took place. There are drawbacks with such a run-in period: increase in study costs, prolongation of the study and underestimation of adverse reactions. However, given the detrimental impact of study withdrawals, a run-in period, which does not exclude participants for reasons other than treatment arm non-compliance, may be worth further consideration for clinical studies.

Whilst online recruitment is effective, remote follow-up strategies do not reduce the loss to follow-up [44]. There are many reasons for loss to follow-up: Participants may move, deaths may be unreported, or the participant may decide to no longer take part without informing study staff. In some instances the participant may simply no longer comply with the study protocol and not explicitly withdraw consent. There continues to be confusion regarding when a participant is formally withdrawn [45], but if the withdrawal of consent is misinterpreted by the study investigator, valuable data may be lost. The most common reasons cited for continuing participation in research are 24-h access to nurses, better treatment of care and a desire to help others [46, 47]. Retention in a clinical trial appears to depend on expert medical care, supportive staff-participant relationships, involvement with clinically and scientifically meaningful research, and management of participant expectations [48]. The extent to which these issues impact the results of TIME remains to be seen.

### Email and telephone interactions

Individuals interested in taking part in the TIME study were able to contact study staff by a Freephone number, by email or by letter. To better understand the resource allocation required for a study using this methodology, we logged all telephone and email contacts for 1 month whilst recruitment was actively being pursued. During a 4-week period in June 2016, 3005 people registered with the study website. Of those, 1860 (62%) went on to consent, complete enrolment and be randomised to a time of dosing. There were 1090 queries to the department about the study during this time: 457 (42%) telephone calls, 522 (48%) emails, and 108 (10%) queries submitted using an online support tool on the study website. There were three additional queries where the method of contact was not recorded. The content of the queries varied: 116 (11%) were IT problems, 199 (18%) were clinically related questions, 701 (64%) were administrative or general enquiries, 64 (6%) were queries about withdrawing from the study, and 10 were complaints about study processes (Table 2).

**Table 2:** Participant contact methods and enquiries for June 2016

	Telephone	Email	Study website	Other
IT query	65	43	6	2
Clinical query	48	100	51	–
Administrative query	332	321	47	1
Withdrawal query	9	54	1	–
Complaint	3	4	3	–

*IT* Information technology

This exercise was repeated in November 2016, when active recruitment had ceased. During this month, 61 individuals registered with the study website, 21 of whom went on to be randomised. There were 401 queries to the department: 300 (75%) by email, 54 (13%) by phone and 57 (14%) via the online support tool. Of these, there were 8 (2%) IT issues, 48 (12%) clinical queries, 302 (75%) administrative or general enquiries, 42 (10%) queries on how to withdraw and 1 complaint (Table 3).

**Table 3:** Participant contact methods and enquiries for November 2016

	Telephone	Email	Study website	Other
IT query	1	6	1	–
Clinical query	4	32	12	–
Administrative query	45	224	33	–
Withdrawal query	4	37	1	–
Complaint	–	1	–	–

*IT* Information technology

### Email and telephone interactions review

The literature on email use in clinical studies is positive, with reports of it being used effectively in research [6–14]. The amount of resource that email and telephone enquiries required was large during the TIME recruitment phase. This is one area that should not be underestimated by researchers when planning study resources. The emails themselves became a key form of data. It became clear during the study that they potentially provide richly contextual prospective records, and this remains an area that is under-researched. Emails often recorded the context of volunteering and the motivations and priorities of volunteers. The volume of both emails and telephone calls highlighted the ‘invisible work’ of research staff during what are typically considered to be standard administrative tasks [49]. One area of concern highlighted by the literature was the lack of a standard process on how to store emails sent by participants containing confidential data. More attention should be paid to confidentiality, documentation and costing when using email communication in order to optimize its use [50].

## Discussion

This work has several limitations. Primarily, the literature review was conducted exclusively using the PubMed library with generic search terms. Analysis of recruitment strategies were limited to methods employed by the Medicines Monitoring Unit (MEMO), and authors were unable to analyse recruitment methods used by the supporting research networks.

Adherence to allocated treatment is under-reported in trial publications. This may be due to a concern about undermining study results. Researchers have attempted to improve participant adherence with varying success. However, loss to follow-up continues to be a problem. Lack of general participant understanding of research objectives and methodologies may contribute to this. There still appears to be confusion among both participants and researchers about when lack of adherence qualifies as withdrawal from the study or as loss to follow-up. Further work is necessary in clarifying study documentation to understand the differences and how to report these phenomena.

Further research is necessary regarding the now common use of email correspondence between researchers and participants. The proclivity to engage in email use in studies suggests that guidelines on best practices and efforts to secure patient confidentiality should be examined expeditiously.

## Conclusions

The TIME study has successfully recruited more than 21,000 participants over a 2-year period. The recruitment methods used have proven successful and cost-effective in comparison to similar-sized studies. The total cost of this major trial is therefore an order of magnitude lower than the cost of a typical commercial clinic-based study. On the basis of experiences in the TIME study, as well as comparisons drawn from the literature, an online methodology and the use of EDC are viable for conducting clinical research. Numerous strategies should be incorporated to improve recruitment and optimise the process, and all available strategies should be used to improve study retention.
