# Correction to: The Treatment In Morning versus Evening (TIME) study: analysis of recruitment, follow-up and retention rates post-recruitment

**DOI:** 10.1186/s13063-021-05581-2

**Published:** 2021-09-07

**Authors:** David A. Rorie, Robert W. V. Flynn, Isla S. Mackenzie, Thomas M. MacDonald, Amy Rogers

**Affiliations:** 1 Division of Molecular and Clinical Medicine, University of Dundee, Ninewells Hospital and Medical School, Dundee, DD1 9SY UK; 2 Medicines Monitoring Unit, University of Dundee, Ninewells Hospital and Medical School, Dundee, DD1 9SY UK

**Correction to: Rorie et al. Trials 18, 557 (2017)**

**http://orcid.org/10.1186/s13063-017-2318-4**

Following the publication of the original article [1], the authors identified 12 participant accounts that should not have been included in the initial reported participant numbers.

The correct number of consented participants for the TIME study are 21,104.

Corrected Table 1 is also presented below:

**Table 1:** TIME registration statistics. How participant heard about study

Recruitment method	Registrations (***n*** = 31,052)	Randomised (***n*** = 21,095)
Radio	73	65
Friends/relatives	97	62
Web search	101	58
From GP	22,894	16,066
MEMO website	105	46
Practice poster	271	170
TIME video (YouTube)	13	6
Medscape	8	3
BHF Heart Matters	338	253
Pharmacy	27	16
Newspaper	18	9
Hospital or clinic	944	619
Biobank	5358	3243
GoShare	55	34
Other	667	409
No Answer provided	83	36
